# Supplementary material for: Osmotic Stress Interferes with DNA Damage Response and H2AX Phosphorylation in Human Keratinocytes
Source: Cells. 2022 Mar 11;11(6):959. doi: 10.3390/cells11060959 (PMC8946833; doi:10.3390/cells11060959)
Supplement: Supplementary file 1 [file cells-11-00959-s001.zip › cells-1610275-supplementary.pdf]

Supplementary Material

Supplementary Text (Material & Methods)

Western Blot

Cells were lysed in lysis buffer (6 M urea (Carl Roth, Germany), 2 M thiourea (Carl Roth, Germany), 50 mM tris (Carl Roth, Germany)) and then centrifuged for 15000 rpm for 15 min. Protein extracts are in the supernatant, which was transferred into a new tube. Concentration was determined using the PierceTM 660 nm Protein-Assay Kit (ThermoFisher Scientific, Germany). 10 µg of each lysate was resolved by sodium dodecyl sulfate-polyacrylamide gel electrophoresis followed by wet electrotransfer on nitrocellulose membrane (AmershamTM Protran Premium, Netherland). Blocking was performed in tris-buffered saline with 0.1% Tween 20 (Carl Roth, Germany) (TBS-T) and 5% nonfat dry milk (Carl Roth, Germany) for at least 1 h at room temperature or overnight at 4°C. After one washing, the membrane was incubated for at least 2h in TBS-T with 5% milk with the primary antibody. Primary antibodies used were monoclonal antibodies, which specifically recognize the phosphorylated form of the MAPK p38 (Phospho-p38 MAPK (Thr180/Tyr182) Rabbit mAb (Cell Signaling, Germany)) and GAPDH (anti GAPDH, sc-47724, Santa Cruz Biotechnology, Germany). After four washings in TBS-T, the membrane was incubated for at least 2 h at room temperature with the peroxidase-conjugated secondary antibody and then visualized using chemiluminescence reagents (WesternSure®, LI-COR Biosciences, Germany) with the C-DiGit Blot Scanner (LI-COR Biosciences, Germany).

Supplementary Figures

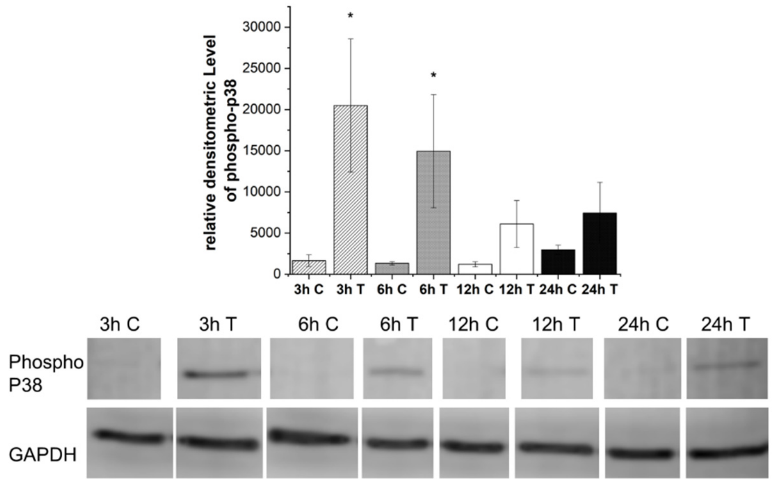

Figure S1. Western Blot analysis of p38 phosphorylation under hyperosmotic conditions ($n = 3$). (A) Densitometric Analyses of phosphorylated p38 in Western Blots from keratinocytes which were treated (T) with 200 mM NaCl or untreated (C) at time points 3, 6, 12 and 24 hours after treatment start, * = $p < 0.05$ B) Representative Western Blot of treated and untreated keratinocytes.

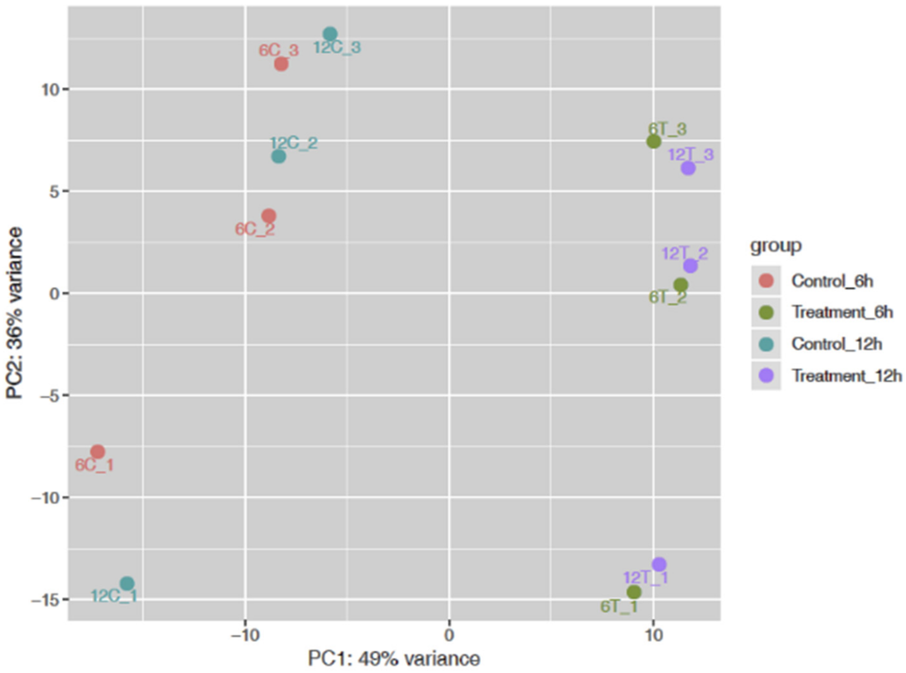

Figure S2. Principle-Component-Analysis of transcriptomic data ($n = 3$) from keratinocytes treated with 200 mM NaCl (Treatment) or left untreated (Control) for 6 and 12 hours.

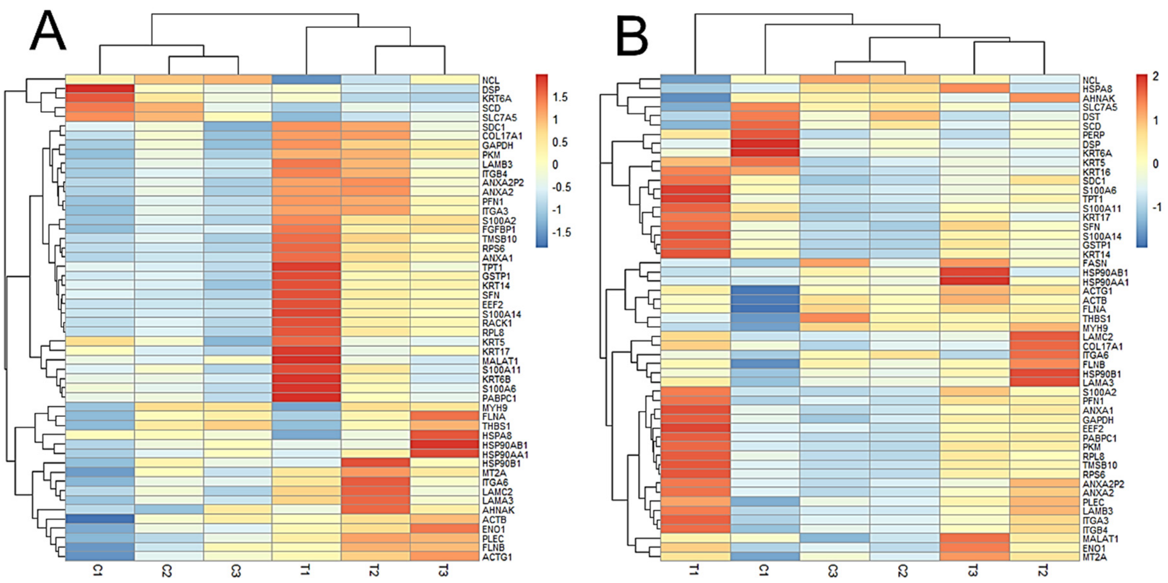

Figure S3. Heatmaps of count data (*n* = 3) after (**A**) six or (**B**) twelve hours of 200 mM NaCl treatment (c = control, t = treatment) created with pHeatmap package, rowsums > 150000, rows were scaled according to the following formula: (x - mean(x)) / sd(x).

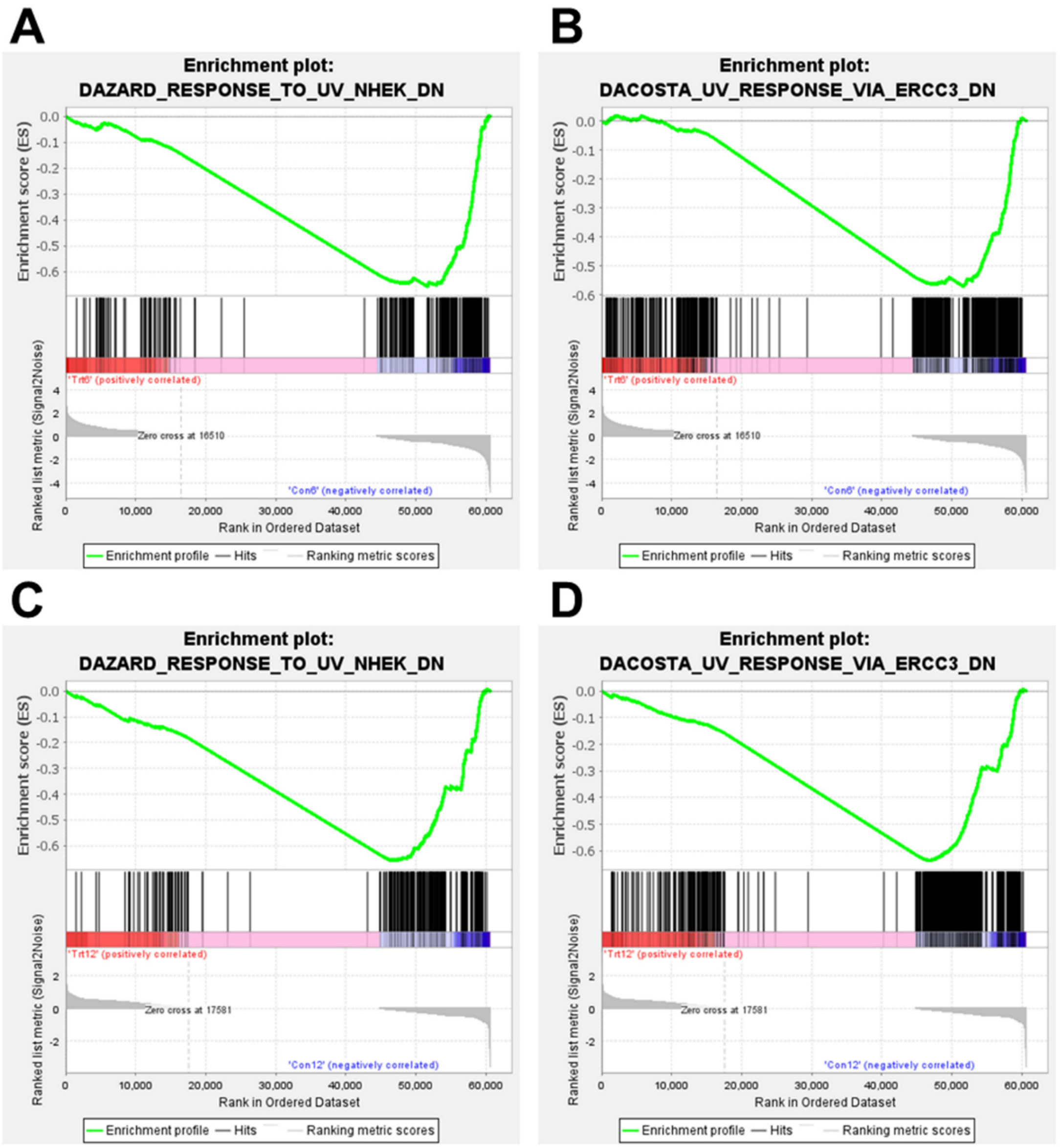

Figure S4. GSEA analysis. GSEA Enrichment plots of dazard (**A**,**C**; NES of -2.02 and -2.16) and dacosta (**B**,**D**; NES of -1.85 and -2.22) gene sets negatively enriched in keratinocytes treated with 200 mM NaCl for 6 (Trt6; **A**,**B**) or 12 (Trt12; **C**,**D**) hours (each $n = 3$), the green curve corresponds to the ES (enrichment score) curve, which is the running sum of the weighted enrichment score obtained from GSEA software, FDR <25%.

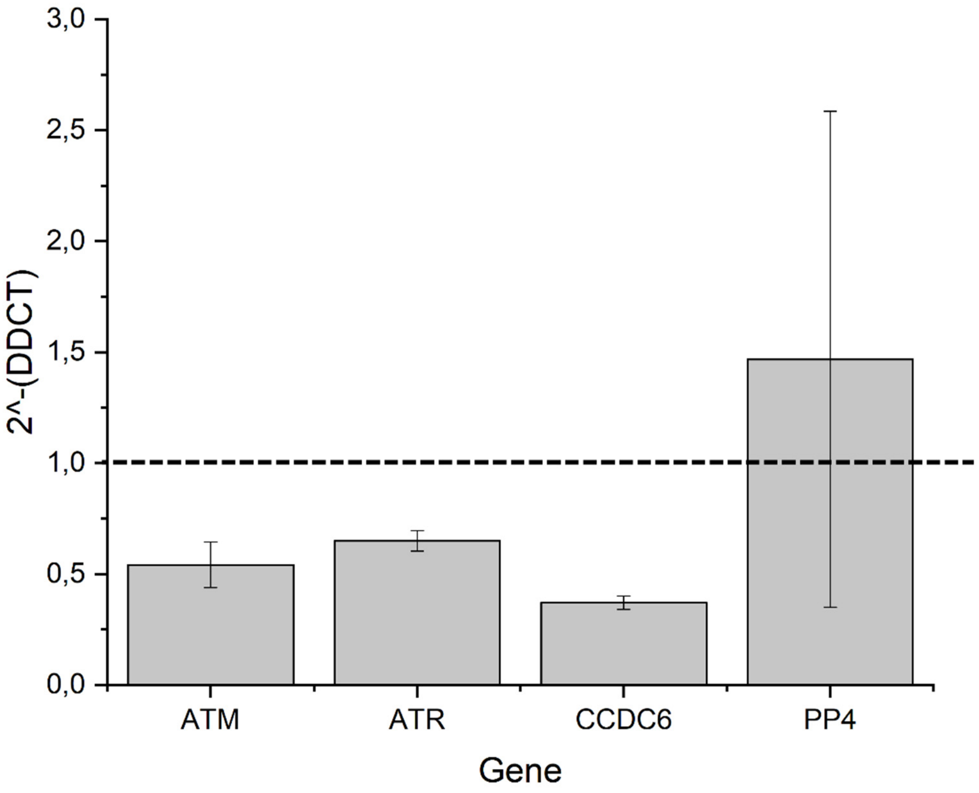

Figure S5. qPCR validation of sequencing data. $2^{-(\Delta\Delta Ct)}$ of the gene expression after 6 hours of 200 mM NaCl treatment (*n* = 3); A reduction in the expression due to treatment ($2^{-(\Delta\Delta Ct}$ <1) can be seen for ATM, ATR and CCDC6, whereas PP4 shows an slight increase in gene expression with higher variance.

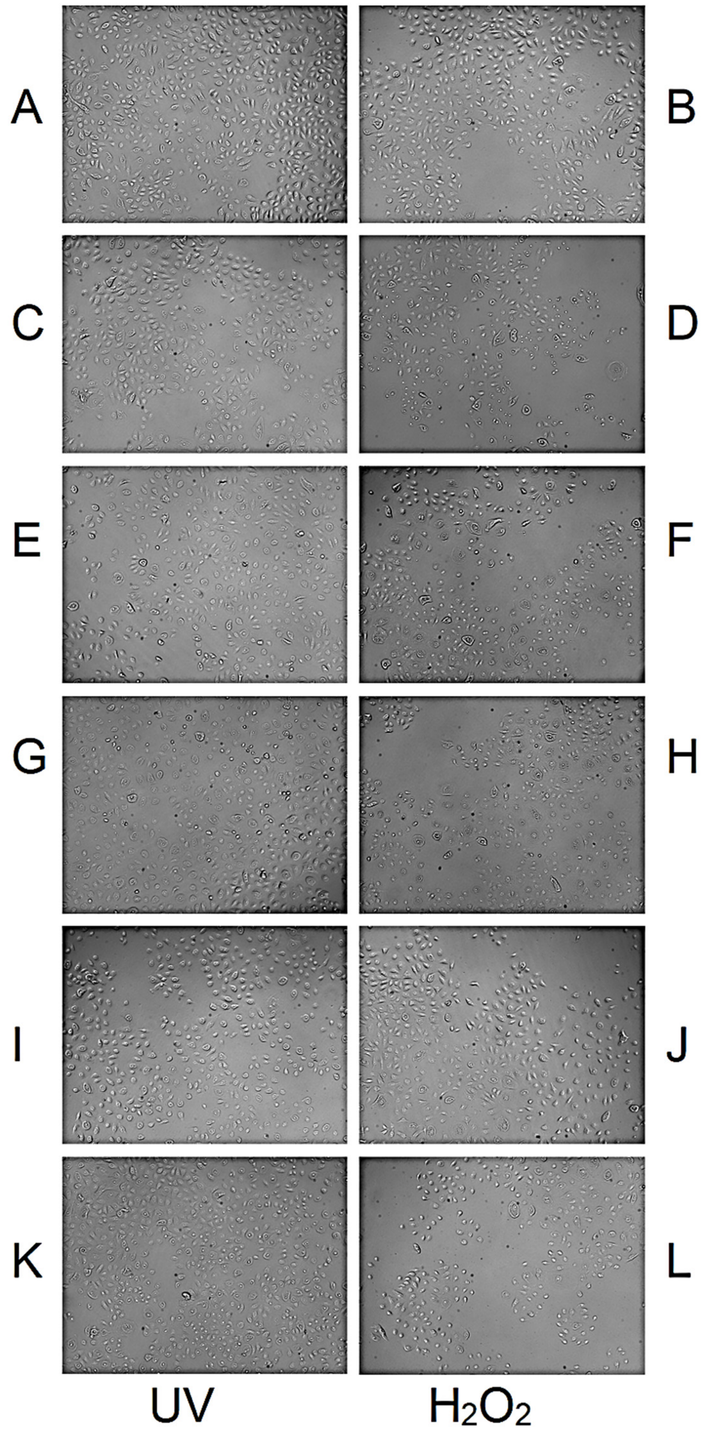

Figure S6. Cell Survival after Costress Experiments (*n* = 1) . Left side shows the treatment with UV (10 min 75 W), right side shows the treatment with H2O2 (300 µM 10 min); A, B Cells were left untreated (no NaCl, no UV/H2O2), C, D Cells are treated with UV/H2O2 with no previously NaCl treatment; E, F Cells are treated with 200mM NaCl for 6 hours; G, H Cells are incubated with 200 mM NaCl for 6 hours and treated afterwards with UV/H2O2; I, J Cells are treated with 100mM NaCl for 6 hours; K, L Cells are incubated with 100 mM NaCl for 6 hours and treated afterwards with UV/H2O2.

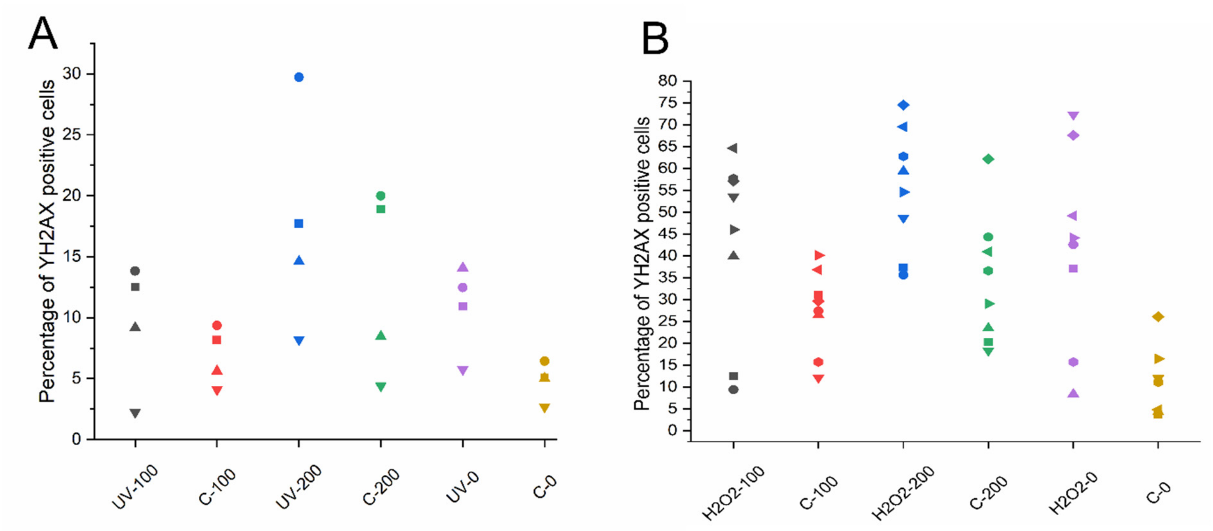

Figure S7. Percentages of γH2AX positive cells in co-stress experiments (**A**) Percentages after UV radiation (n = 4) (**B**) percentages after H2O2 treatment (n = 8).
